# Enhanced sulfidation xanthate flotation of malachite using ammonium ions as activator

**DOI:** 10.1038/s41598-017-02136-x

**Published:** 2017-05-18

**Authors:** Dandan Wu, Wenhui Ma, Yingbo Mao, Jiushuai Deng, Shuming Wen

**Affiliations:** State Key Laboratory of Complex Nonferrous Metal Resources Clean Utilization, Kunming University of Science and Technology, Kunming, 650093 P.R. China

## Abstract

In this study, ammonium ion was used to enhance the sulfidation flotation of malachite. The effect of ammonium ion on the sulfidation flotation of malachite was investigated using microflotation test, inductively coupled plasma (ICP) analysis, zeta potential measurements, and scanning electron microscope analysis (SEM). The results of microflotation test show that the addition of sodium sulfide and ammonium sulfate resulted in better sulfidation than the addition of sodium sulfide alone. The results of ICP analysis indicate that the dissolution of enhanced sulfurized malachite surface is significantly decreased. Zeta potential measurements indicate that a smaller isoelectric point value and a large number of copper-sulfide films formed on the malachite surface by enhancing sulfidation resulted in a large amount of sodium butyl xanthate absorbed onto the enhanced sulfurized malachite surface. EDS semi-quantitative analysis and XPS analysis show that malachite was easily sulfurized by sodium sulfide with ammonium ion. These results show that the addition of ammonium ion plays a significant role in the sulfidation of malachite and results in improved flotation performance.

## Introduction

Copper is the most prevalent and valuable metals used in the industry. Owing to its high conductivity, copper is used mostly in the electrical and electronics industries^1–4^. In nature, copper mainly exists in the form of sulfide ore and oxidized ore^5^, and this form is an important part of copper resources. Malachite is a typical representation of copper oxide ore, and approximately 30% of the copper is extracted from oxidized copper ore worldwide. The increasing copper demand has facilitated the inevitable exploitation and utilization of copper oxide ore. Achieving high combination rate and high clay content are difficult for copper oxide ore because of its complicated formation process; this complicated process also impedes the production of a variety of copper mineral symbiosis with better technical and economic indicators; moreover, copper recovery is generally less than 75%. Therefore, the efficient utilization of copper oxide ore from copper resources is an important subject of research.

Flotation is a method which is commonly used for recovering copper oxide ores. It is divided into sulfidation flotation and direct flotation. Direct flotation of copper oxide ores has been extensively studied. They selected fatty acids, fatty amines, petroleum sulfonates, and hydroxamic as flotation collectors for the direct recovery of copper oxide minerals without sulfidation treatments^6–11^. All of these collectors have shown promise in laboratory tests but limited success when applied to actual plant situations^9^.

Sulfidation xanthate flotation is the most widely used method for dealing with copper oxide ore^9, 12, 13^. Sodium sulfide can promote the flotability of oxide copper ore^14^, resulting in the considerable increase of research attention^12, 13^. Oxide mineral surface has an ionic bond, such that the polarization of water molecules forms a firm hydration film through electrostatic attraction; it also possesses a hydrophilic surface, and using the film hydration effect on mineral surfaces to obtain collectors, such as xanthate, is difficult^15, 16^. The sulfidation process converts the oxide mineral surface using Na_2_S, whereas the adsorption of S^2−^ with HS^−^ ions on the oxide mineral surfaces generates a metal sulfide film in favor of oxide mineral flotation^17^. Moreover, an inadequate amount of sulfidation reagents cause a restricted improvement to copper flotation recoveries; excessive sulfidation reagents present inhibitory effects on copper oxide ore flotation^9, 10^. In addition, the generated copper sulfide film is incompact and easily falls off when strongly stirred. Therefore, eliminating the excess harmful effects of sulfidation reagents and enhancing the sulfidation of the surface malachite are crucial to the sulfidation xanthate flotation of copper oxide ore.

Ammonium salt is mainly used in the leaching of oxidized ore, especially malachite^18–21^. The ammonium ion exhibits two effects on the leaching of malachite. (1) For the dissolution effect, the ammonium salt can continuously hydrolyze with water, which can consume carbonate ions and hydroxyl ions of malachite and release the copper ions. (2) For the complexation effect, dissolved copper ions and ammonium ions are combined into complex ions of copper and ammonia. Therefore, the two characteristics can be used in the sulfidation xanthate flotation of malachite, and a small amount of ammonium ion can be added to expose the malachite flotation surface to a large amount of copper ions. The exposed copper ions on the malachite mineral surface increase the activity of malachite minerals and promote the flotation of malachite.

In the previous study, Zhang^22^ found that ammonium sulfate in the process played a significant role in promoting the curing, which mainly contains: (1) catalytic effect. The addition of ammonium sulfate greatly accelerated the speed of malachite sulfidation reaction and promoted the reaction, thus avoiding the inhibition of excess sulfur Ions; (2) stability effect. After adding ammonium sulfate, it will make the produced sulfide film on the malachite surface more stable; (3) hydrophobic effect. Ammonium sulfate improves the adsorption rate and adsorption quantity of xanthate on the malachite surface, thus making the malachite more hydrophobic. They confirmed that ammonium ion can promote the sulfidation flotation of malachite based on flotation experiments. However, no other test method can verify this result. The mechanism of ammonium ions on sulfide flotation of malachite was studied.

In this study, the enhancing effect of ammonium sulfate on the sulfidation of malachite was investigated through microflotation tests, inductively coupled plasma (ICP) analysis, zeta potential measurements, scanning electron microscopy (SEM) studies and X-ray photoelectron spectral (XPS) analysis.

## Results and Discussion

### Microflotation studies

**Figure 1:**
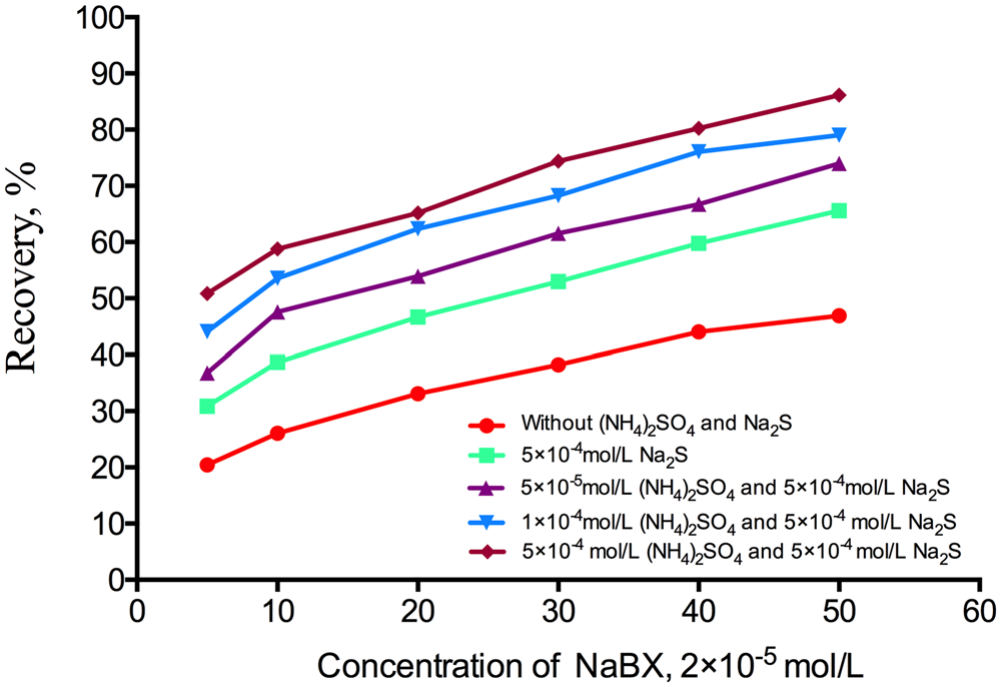
Flotation recovery of malachite as a function of NaBX concentration in the absence or presence of different concentrations of (NH_4_)_2_SO_4_ using 5 × 10^−4^ mol/L Na_2_S·9H_2_O as a sulfidizing agent.

The flotation recovery of malachite as a function of sodium butyl xanthate (NaBX) concentration in the absence or presence of different ammonium sulfate concentrations with 5 × 10^−4^ mol/L Na_2_S·9H_2_O as a sulfidizing agent is shown in Fig. 1. With NaBX as the collector, malachite floatability was weak without sulfidation prior to flotation, and the flotation recoveries of malachite were 20.42% at 2 × 10^−5^ mol/L concentration of NaBX and 51.36% at 1 × 10^−3^ mol/L concentration of NaBX. After adding 5 × 10^−4^ mol/L Na_2_S·9H_2_O as a sulfidizing agent, the recovery of malachite considerably increased with increasing NaBX concentration. Moreover, the addition of 5 × 10^−5^ mol/L ammonium sulfate as the activator considerably increased the flotation recovery of malachite at the same concentration of Na_2_S·9H_2_O and NaBX. Under the experimental conditions above, the flotation recovery of malachite increased considerably with ammonium sulfate concentration. The flotation recovery of malachite increased from 78.28% to 90.86% when the ammonium sulfate concentration was increased from 5 × 10^−5^ mol/L to 5 × 10^−4^ mol/L using 1 × 10^−3^ mol/L NaBX as the collector. The addition of ammonium salts is an effective way for enhanced sulfidation on the sulfide-xanthate flotation of copper oxide^22^. We obtained the same result as that of ref. 22, i.e., ammonium ions can promote the sulfidation of malachite and can substantially improve the sulfidation flotation of malachite. Prior to the addition of the sulfidizing agent, the addition of the inorganic ammonium salt to the slurry solution can not only enhance the activation of HS^−^ ions but also eliminate or reduce the adverse effects of residual HS^−^ ions. This can reduce the amount of sodium sulfide and make the sulfide film more compact and stable, which is conducive to improving the recovery and grade of copper oxide ore. The results show that the addition of ammonium sulfate played a significant role in the sulfidation of malachite, and this sulfidation could facilitate its flotation and improve flotation performance.

The effect of the addition of ammonium salt on the pH value and flotation effect when the dosage of NaBX is 1 × 10^−3^ mol/L under different flotation conditions is shown in Table 1. The results showed that the pH value of the solution system during 7, and the best flotation efficiency was 51.36%, which is not conducive to the flotation of malachite in the range of the pH value. After adding 5 × 10^−4^ mol/L Na_2_S, the pH value of the system was changed 11.2 because of the hydrolysis of sodium sulfide. The pH value is a more favorable condition for the flotation of malachite compared with that without any reagent. When using different concentrations of ammonium sulfate as the activator, the pH value of the system is more stable than that with the addition of sodium sulfide. When the concentration of ammonium sulfate is between 5 × 10^−5^ mol/L and 5 × 10^−4^ mol/L, the pH value of the solution decreased. However, the range of change was small, and the recovery rate of malachite flotation increased from 78.28% to 90.86%. The pH value of the ammonium salt is stable in the flotation system, that is, the stability of the flotation environment pH value is beneficial to the stability of the zeta potential of the malachite surface, thereby promoting the stability of reagent adsorption. On the other hand, it was suitable for the stability of the flotation system and ensured the stability of flotation.

**Table 1:** Effect of pH on Microflotation of malachite.

Test condition	pH values during the experiments	Recovery, %
Without (NH_4_)_2_SO_4_ and Na_2_S	7	51.36
5 × 10^−4^ mol/L Na_2_S	11.2	68.08
5 × 10^−5^ mol/L(NH_4_)_2_SO_4_ and 5 × 10^−4^ mol/L Na_2_S	10.8	78.28
1 × 10^−4^ mol/L (NH_4_)_2_SO_4_ and 5 × 10^−4^ mol/L Na_2_S	10.5	84.11
5 × 10^−4^ mol/L (NH_4_)_2_SO_4_ and 5 × 10^−4^ mol/L Na_2_S	10	90.86

### Dissolution tests of malachite under different conditions by ICP analysis

The dissolution tests of malachite under different conditions are shown in Table 2. No trend was observed in the results of the four separate group tests, but some rules were still found among different groups. The dissolution of malachite in deionized water showed an irregular trend in volatility within 60 min, and the copper ion concentration in deionized water was between 1.12 and 3.22 mg/L. However, time extension did not have much effect on this dissolution. In the second test, we determined the effect of ammonium sulfate on the dissolution of malachite in deionized water and obtained the same results as that no significant change was observed without ammonium sulfate. However, we found that the copper ion concentration was more than twice that of the first group’s data, because ammonium sulfate is a strong acid that becomes a weak alkali when it hydrolyzes. The following ammonium sulfate hydrolytic reaction equations will occur in the solution.

1 $${({{\rm{NH}}}_{4})}_{2}{{\rm{SO}}}_{4}\rightleftharpoons 2{{{\rm{NH}}}_{4}}^{+}+{{{\rm{SO}}}_{4}}^{2-}$$

2 $${{{\rm{NH}}}_{4}}^{+}+{{\rm{H}}}_{2}{\rm{O}}\rightleftharpoons {{\rm{NH}}}_{3}\cdot {{\rm{H}}}_{2}{\rm{O}}+{{\rm{H}}}^{+}$$

**Table 2:** Dissolution and time relationship of analysis results of malachite under different conditions.

Time (min)	Copper ion concentration of deionized water (mg/L)	Copper ion concentration of ammonium sulfate solution (mg/L)	Copper ion concentration of sodium sulfide solution (mg/L)	Copper ion concentration of ammonium sulfate and sodium sulfide solutions (mg/L)
1	1.91	5.23	0.52	0.032
3	2.04	5.89	0.43	0.021
6	1.91	6.01	0.44	0.038
10	3.22	5.75	0.31	0.041
15	2.76	6.21	0.35	0.036
20	1.20	5.54	0.22	0.033
25	2.94	5.71	0.37	0.027
30	2.12	6.22	0.28	0.013
60	1.12	6.11	0.18	0.015

Hydrolysis-generated hydrogen ions can consume the carbonate and hydroxyl ions of malachite, thereby releasing the copper ions. The dissolved copper and ammonium ions are combined into complex ions of copper and ammonia, which can be represented by the following reaction equations.

3 $${{\rm{Cu}}}^{2+}+2{{\rm{NH}}}_{3}\leftrightarrows {\rm{Cu}}{({{\rm{NH}}}_{3})}_{2}{}^{2+}$$

4 $${\rm{Cu}}{({{\rm{NH}}}_{3})}_{2}{}^{2+}+2{{\rm{NH}}}_{3}\leftrightarrows {\rm{Cu}}{({{\rm{NH}}}_{3})}_{4}{}^{2+}$$

We also tested the effect of sodium sulfide on the dissolution of malachite in deionized water; the result was one order smaller than that of the dissolved malachite in deionized water. The CuS produced by the chemical reaction between S^2−^ and dissolve Cu^2+^ could be absorbed on the malachite surface and inhibit the dissolution of malachite. However, S^2−^ would consume Cu^2+^ in the solution and reduce its concentration. Interestingly, when ammonium sulfate and sodium sulfide were added to the dissolution of malachite, the result was two orders smaller than that of the first experiment; three orders smaller than that of the second experiment; and one order smaller than that of the third experiment.

After the addition of sodium sulfide, the following reaction occurs:

5 $${{\rm{Na}}}_{2}{\rm{S}}\rightleftharpoons 2{{\rm{Na}}}^{+}+{{\rm{S}}}^{2-}$$

6 $${{\rm{S}}}^{2-}+{{\rm{H}}}_{2}{\rm{O}}\rightleftharpoons {{\rm{HS}}}^{-}+{{\rm{OH}}}^{-}$$

According to Eqs (1)–(6), the Na^+^ and SO_4_^2−^ in the ammonium sulfate and sodium sulfide solution do not participate in the reaction. The H^+^ in Eq. (2) consumes the OH^−^ in the reaction in Eq. (6), such that the reaction in Eq. (6) proceeds in the positive direction according to the chemical reaction equilibrium. That is, the S^2−^ in the solution is continually hydrolyzed to HS^−^ under the sustained effect of the H^+^ generated by the hydrolysis of ammonium ion.

The chemical reaction between S^2−^ and HS^−^ on the surface of malachite is shown in the following equation.

7 $${{\rm{Cu}}}_{2}{{\rm{CO}}}_{3}{({\rm{OH}})}_{2}({\rm{surf}}\,)+2{{\rm{S}}}^{2-}\rightleftharpoons 2{\rm{CuS}}({\rm{surf}}\,)+{{{\rm{CO}}}_{3}}^{2-}+2{{\rm{OH}}}^{-}$$

8 $${{\rm{Cu}}}_{2}{{\rm{CO}}}_{3}{({\rm{OH}})}_{2}({\rm{surf}}\,)+2{{\rm{HS}}}^{-}\rightleftharpoons 2{\rm{CuS}}({\rm{surf}}\,)+{{\rm{CO}}}_{2}+{{\rm{H}}}_{2}{\rm{O}}+2{{\rm{OH}}}^{-}$$

${{\rm{\Delta }}}_{f}{G}_{m}^{{\rm{\Theta }}}$ in Equation (7) is more negative than that in Equation (8). Therefore, Equation (8) is more likely to occur than Equation (7). In particular, HS^−^ becomes more easily absorbed on the surface of malachite than S^2−^, which in turn will consume a large amount of HS^−^ and move Eq. (6) in the positive direction. In Eqs (1)–(8), a large amount of CuS are produced because of the increasing HS^−^ in the solution. HS^−^ was more easily absorbed on the surface of malachite than S^2−^; therefore, more CuS was produced on the surface of the enhanced and sulfurized malachite. By reacting with ammonium ion, the malachite became easily sulfurized by sodium sulfide. In addition, the enhanced and sulfurized malachite generated a large amount of CuS, suggesting that the ammonium ion enhanced the effect of sulfide on the malachite.

The solubility product constant of CuS (6.3 × 10^−36^) was far less than that of CuCO_3_ (2.34 × 10^−10^) and Cu(OH)_2_ (4.8 × 10^−20^), such that dissolving CuS in the pulp solution was difficult. This phenomenon suggests that the CuS on the surface of malachite inhibited the dissolution of malachite. Therefore, the concentration of Cu^2+^ in the malachite dissolved in ammonium sulfate, and sodium sulfide solution was much less than that in the three other experiments. The concentration of Cu^2+^ in the enhanced sulfidation experiment was one order smaller than that of the direct sulfidation experiment. This finding suggests that a large amount of CuS were produced on the surface of the enhanced and sulfurized malachite.

### Zeta potential measurements

The zeta potential is a technique used to investigate the surface charge of the mineral/solution interface; this technique has been widely applied to interpret the modification of flotation performance and the trend of flotation efficiency caused by the presence of reagents^23–25^. The zeta potential can reflect the surface charge state of the charged particles and is affected by the surface potential of minerals, type of exchange ions, solution concentration, ionic composition, dielectric constant of the solution, pH, temperature, and other factors^26^. Studying the relationship between the zeta potentials of electrical double layers on particle surfaces and the adsorption properties of a surface contributes to the exploration of chemical processes on the particle surface and ion exchange law of particles^27^.

**Figure 2:**
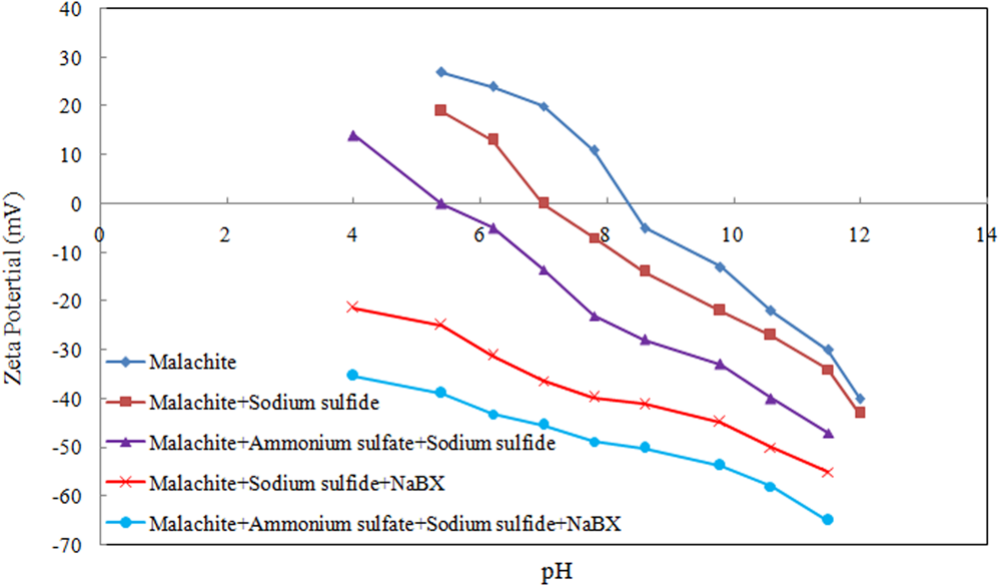
Zeta potentials of malachite as a function of pH in the absence or presence of Na_2_S, (NH_4_)_2_SO_4_, and NaBX.

The zeta potentials of malachite as a function of pH in the absence or presence of Na_2_S, (NH_4_)_2_SO_4_, and NaBX are shown in Fig. 2. The isoelectric point (IEP) of malachite in this experiment was pH = 8.2, while the malachite surface was negatively charged when pH > 8.2 and positively charged when pH < 8.2. After the addition of Na_2_S, the pH_IEP_ of malachite decreased from 8.2 to 6.9. This phenomenon is caused by the chemical reaction of S^2−^ and Cu^2+^ during malachite dissolution. The copper-sulfide film was adsorbed on the malachite surface, which significantly reduced the Cu^2+^ present. The effect of ammonium sulfate on malachite sulfidation was also tested by the zeta potential. The test results show that the addition of sodium sulfide and ammonium sulfate in the solution resulted in the pH_IEP_ value smaller than that after the addition of sodium sulfide alone. In particular, the pH_IEP_ value is decreased to 5.4. The experiment shows that the addition of sodium sulfide and ammonium sulfate resulted in better sulfidation and formed a large number of copper-sulfide films on the malachite surface than the addition of sodium sulfide alone. We also tested the effect of NaBX on ammonium sulfate and sodium sulfide solutions by measuring the zeta potentials. The results indicate more negative potential value than that of the previously mentioned tests. This phenomenon shows that the amount of NaBX absorbed onto the sulfurized malachite surface increased in the presence of ammonium sulfate. This finding agrees with the results of the microflotation and dissolution tests of malachite.

### SEM-EDS analysis

**Figure 3:**
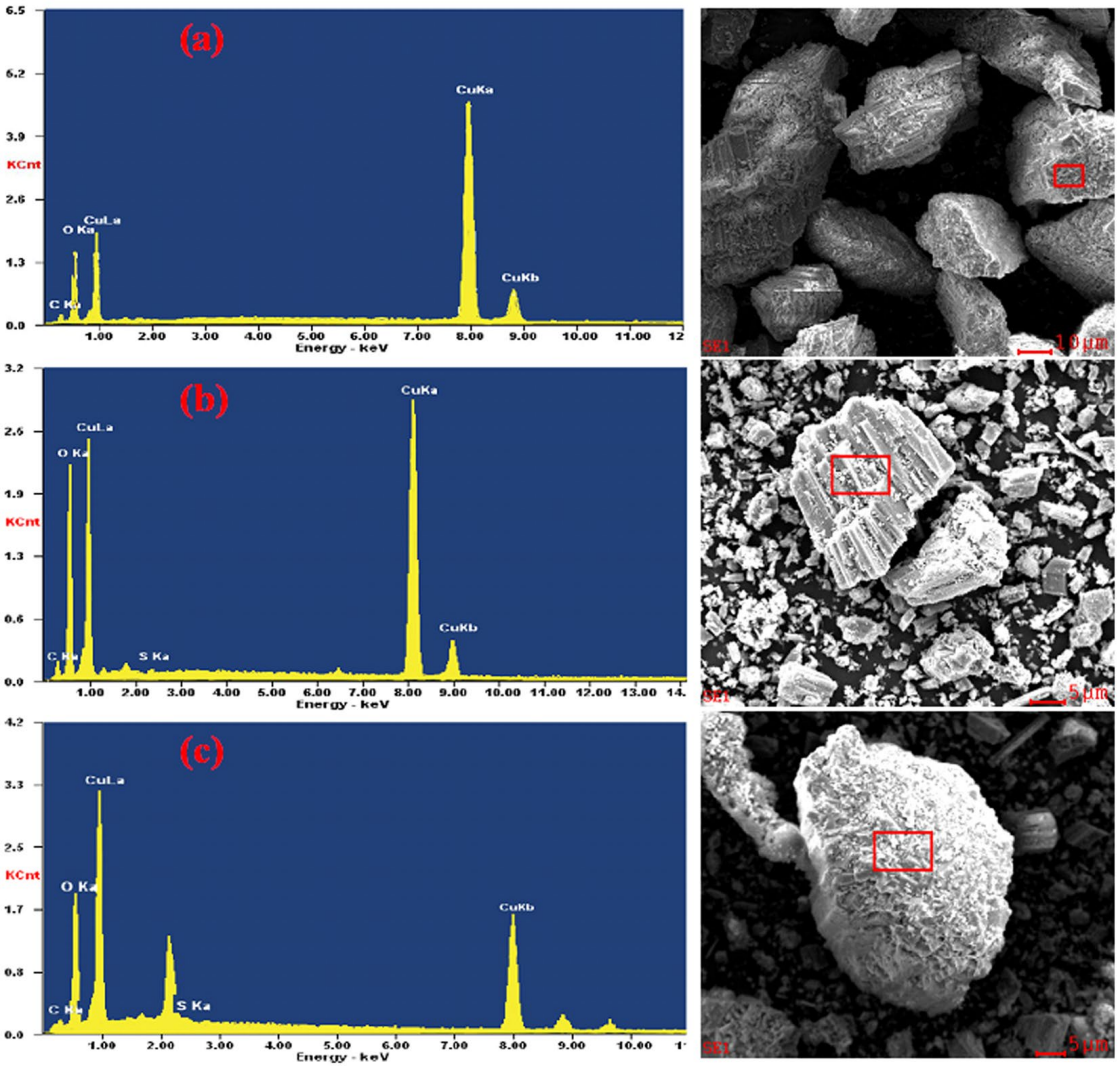
EDS spectra of malachite samples: (**a**) original malachite; (**b**) after sulfidation; (**c**) after enhanced sulfidation.

To further prove the activation effect of ammonium ions on the sulfidation flotation of malachite, EDS analysis was conducted on the surface of the original malachite, after sulfidation and enhanced sulfidation. The results are shown in Fig. 3a–c. The result in Table 3 shows the content of the EDS spectrum element in semi-quantitative analysis results of the three samples. The electron energy spectra in Fig. 3a contain Cu, C, and O peaks. We did not detect an N peak because the ammonium ion promoted the dissolution of malachite. On the contrary, Cu^2+^ reacted with NH_4_^+^ to form the copper ammonia complex ion in the aqueous solution. The electron energy spectrum in Fig. 3b shows an S peak in addition to the C, O, and Cu peaks. Conversely, the semi-quantitative analysis shows that the content of S was 1.86%, suggesting that S reacted with malachite and attached to the mineral surface. The electron energy spectrum in Fig. 3c shows C, O, Cu, and S peaks. Similar to the previous results, no N peak was detected. The semi-quantitative analysis shows that the concentration of S atom was 6.63%. Compared with the direct sulfidation test, the concentration of S atom increased from 1.86% to 6.63%, but the concentration of O atom decreased from 46.21% to 39.84%. Therefore, the amount of copper sulfide films generated on the surface of the enhanced sulfidation malachite was increased. Owing to the evidently enhanced sulfidation of ammonium ion, the malachite was easily absorbed by sodium sulfide after reacting with ammonium ion.

**Table 3:** Content of EDS spectrum elements in semi-quantitative analysis results.

	Element	Mass Concentration %	Atomic Concentrations %
(a)	*CK*	13.01	25.66
	*OK*	31.31	49.97
	*CuK*	55.68	24.37
(c)	*CK*	09.53	21.02
	*OK*	28.28	46.21
	*SK*	01.22	01.86
	*CuK*	60.97	30.91
(d)	*CK*	07.13	18.32
	*OK*	21.16	39.84
	*SK*	04.78	06.63
	*CuK*	66.93	35.21

### XPS analysis

**Figure 4:**
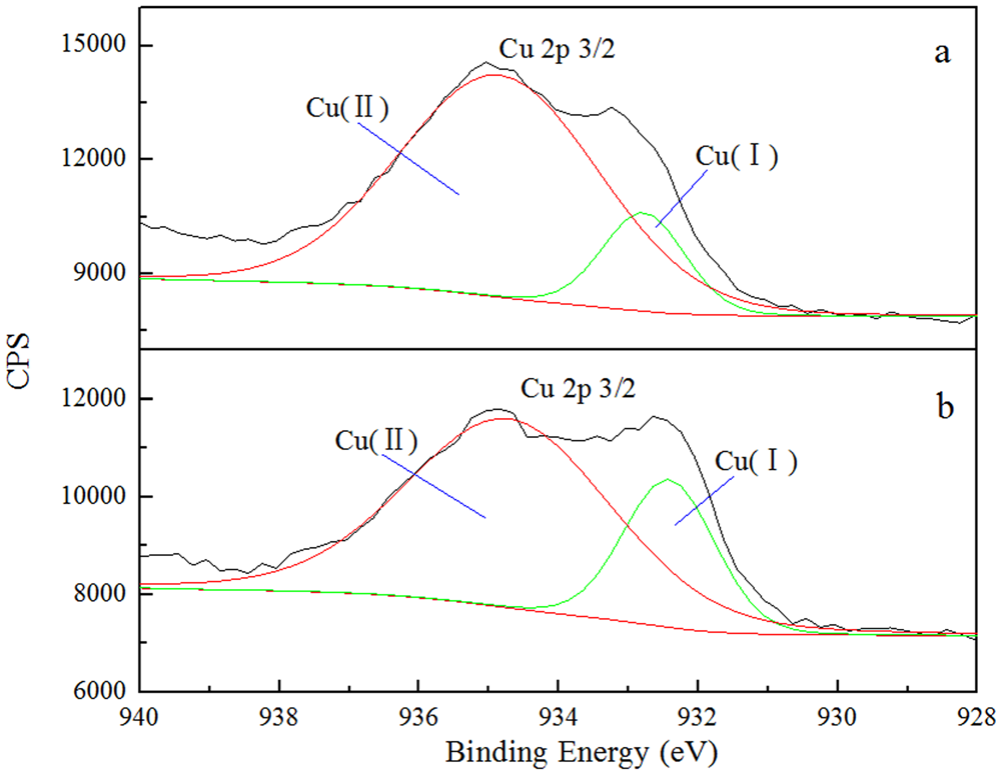
Cu 2p3/2 spectra of malachite after (**a**) sulfidation and (**b**) enhanced sulfidation.

**Figure 5:**
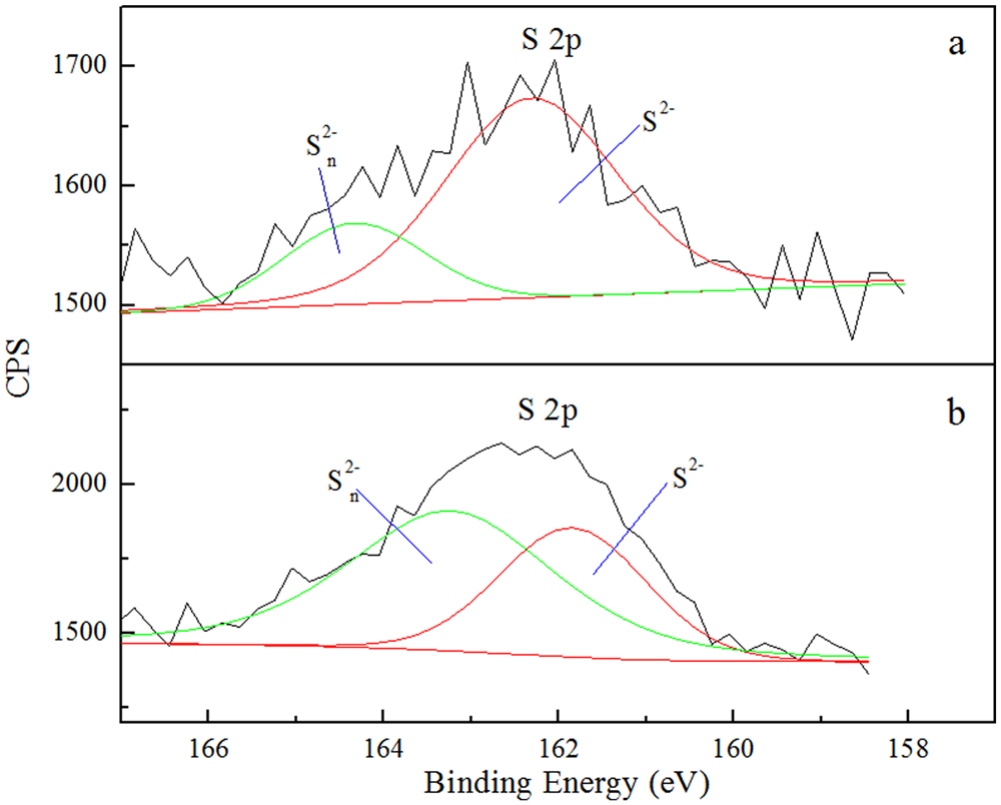
S 2p spectra of malachite after (**a**) sulfidation and (**b**) enhanced sulfidation.

X-ray photoelectron spectroscopy (XPS) is an important surface analysis method that can be used to study the elemental composition and chemical valence of solid surface. An XPS analysis was performed on sulfurized and enhanced sulfurized malachite. Figure 4 showed the Cu 2p3/2 spectra of malachite after sulfidation and enhanced sulfidation. The XPS spectra of sulfurized and enhanced sulfurized malachite show two peaks for Cu, their binding energies were 932.78 eV, 934.85 eV and 932.42 eV, 934.71 eV, respectively. Cu 2p3/2 signal at 932.4 eV has been reported^28–30^ as being characteristic of Cu (I). Cu 2p3/2 with the binding energy at 934 eV is the characteristic peak of Cu (II)^28^. Figure 5 showed that the S 2p spectra of malachite after sulfidation and enhanced sulfidation. The XPS spectra of sulfurized and enhanced sulfurized malachite show two peaks in the electron energy spectrum of S, which indicates that their binding energy were separately located at 162.30 eV, 164.30 eV and 161.84 eV, 163.23 eV. According to a previous study^31–33^, the S 2p peak with a binding energy that ranges from 161.2 eV to 162.3 eV is the divalent sulfide ion (S^2−^), whereas higher binding energies (162.4 eV to 164.3 eV) relate to the polysulfide ion (S_*n*_^2−^, *n* ≥ 2), which results from the slight oxidation of divalent sulfur on malachite surface. The S peaks suggest the presence of S^2−^ and S_*n*_^2−^ on the malachite surface. Thus, during malachite sulfidation, the copper on malachite surface is present in the form of Cu (I) and Cu (II). Sulfur is present in the form of S^2−^ and S_*n*_^2−^, thereby indicating that the chemical valence of some copper during sulfidation is decreased. Some sulfion is oxidized, and the copper–sulfur compound will be produced during malachite sulfidation may be Cu_x_S_y_. In addition, the ratio of the S 2p3/2 occupied area and the atom concentration on the surface of malachite are 5.03% and 3.81% %, which are higher than the results of direct sulfidation (3.38% and 2.60%). This finding suggests that the degree of sulfur absorption on the surface of the enhanced sulfurized malachite is stronger than that on the surface of direct sulfurized malachite. That is to say, the efficiency of sulfidation in the enhanced sulfurized malachite is much higher, which once again proves that it is easily sulfurized on the surface of the enhanced sulfurized malachite.

## Conclusions

Ammonium ions were used to enhance the sulfurized xanthate flotation of malachite for a good flotation effect. This goal was achieved owing to the increased formation of copper sulfides on the malachite surface, which was characterized by the results of microflotation tests, ICP analysis, zeta potential measurements, SEM-EDS analysis and XPS analysis. On the basis of the results, the following conclusions are obtained:

1. The microflotation test results show that the ammonium ions facilitated the flotation of malachite. The addition of sodium sulfide and ammonium sulfate resulted in better sulfidation than the addition of sodium sulfide alone; in addition, the flotation recovery increased by approximately 27 percentage points.
2. The malachite dissolution tests in different conditions indicate that the concentration of copper ion in the ammonium sulfate solution is nearly doubled compared with that in pure water. When malachite was affected by sodium sulfide, the concentration of copper ion in the solution was reduced by one order of magnitude compared with that in pure water. When malachite was affected by ammonium sulfate and sodium sulfide, the concentration of copper ion in the solution was reduced by two orders of magnitude compared with that in pure water and reduced by one order of magnitude compared with that in the sodium sulfide solution. This result indicates that the dissolution of the enhanced sulfurized malachite surface decreased significantly.
3. Zeta potential measurements indicate that the addition of sodium sulfide and ammonium sulfate resulted in an IEP value smaller than that after the addition of sodium sulfide alone. Moreover, the IEP value is decreased from 6.9 to 5.4. The formation of copper-sulfide films on the malachite surface was increased by enhancing sulfidation, and a large amount of NaBX were absorbed onto the enhanced sulfurized malachite surface.
4. EDS-based semi-quantitative analysis shows that the concentrations of sulfur on the surface of sulfurized and enhanced sulfurized malachite were 1.86% and 6.63%, respectively. Malachite was easily sulfurized by sodium sulfide under the effect of ammonium ions.
5. XPS analysis indicates that the sulfidation and addition of ammonium ions increased the sulfur ions on the sulfurized malachite surface. The ratio of the S 2p3/2 occupied area and the atom concentration on the surface of malachite are 5.03% and 3.81% %, which are higher than the results of direct sulfidation (3.38% and 2.60%).

## Materials and Methods

### Materials and reagents

**Figure 6:**
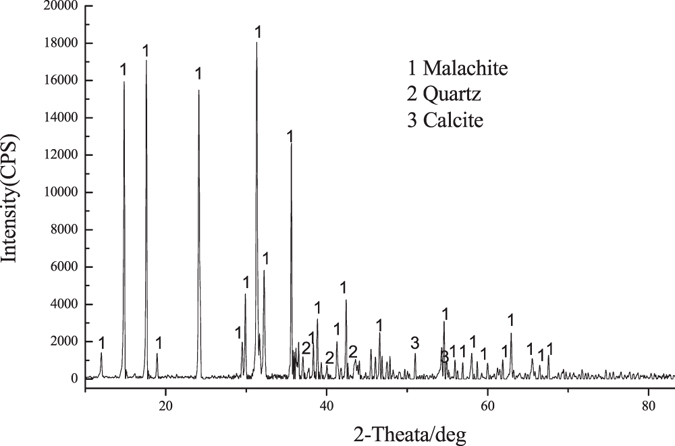
XRD pattern of the original malachite.

Pure malachite samples were obtained from Yunnan Province, China. The ore samples were comminuted in an agate mortar, and coarse fraction (−74 μm to +19 μm) was used in the microflotation tests. Using a Japan Science D/max-R diffractometer apparatus, X-ray diffraction (XRD) experiments were performed with CuKα radiation (*λ* = 1.5406 Å) at an operating voltage of 40 kV and a current of 40 mA. The diffraction angles (2*θ*) ranged from 10° to 80°. The XRD pattern and chemical composition of malachite are shown in Fig. 6 and Table 4, respectively. Figure 5 shows the X-ray analysis results, which reveal that the samples consist of malachite, quartz, and calcite.

**Table 4:** Chemical composition of the pure malachite used in the experiments.

Element	CuO	Fe_2_O_3_	CaO	MgO	Al_2_O_3_	SiO_2_
Content (%)	67.3	0.58	1.03	0.09	0.03	3.49

Ammonium sulfate was selected as the source of ammonium ions. Na_2_S·9H_2_O was added as a sulfidizing agent, and commercial-grade NaBX was used as the collector. All of the reagents used in the tests were analytically pure, except for NaBX. Pure deionized water was used for all experiments.

### Microflotation tests

Microflotation tests were performed using an agitation flotation machine. The mineral suspension was prepared by adding 5.0 g of pure malachite to 40 mL solutions. The ammonium sulfate solutions of various concentrations were first added into the pulp solution for 5 min and added to Na_2_S·9H_2_O solutions as the sulfurizing reagent for 5 min. Subsequently, the pH value of the pulp was adjusted to 10. The pulp was then conditioned with NaBX for 5 min and floated for another 5 min. After the flotation tests, the concentrate and tailings were filtered and dried before being weighed. Flotation recovery was calculated based on the solid weight distribution between the two products.

### Dissolution test of malachite under different conditions by ICP analysis

Pure malachite that is approximately 0.5–1 cm in particle size was cleaned with deionized water. Pure mineral that has been dried was weighed for each experiment and placed into two ball milling jars of similar models. The closed ball milling pots were installed on the impact ball milling instrument (MM400, Retsch, Germany), and vibration frequency and grinding time were set to 900 min^−1^ and 5 min, respectively. The dissolution test was carried out through mechanical stirring in a beaker at different times according to each test sample of 2 g malachite mineral and pulp solution concentration of 5%. The dissolution test was divided into four parts, each lasting for 120 min. Specifically, the malachite mineral was dissolved in deionized water, ammonium sulfate solution, sodium sulfide solution, ammonium sulfate solution, and sodium sulfide solution. The concentration of ammonium sulfate and sodium sulfide was 5 × 10^−4^ mol/L, and each test was conducted in a neutral environment. After dissolution, the supernatant was measured by ICP analysis using a centrifuge (TL-4.7 W, SCI, China) for solid–liquid separation.

### Zeta potential measurements

The zeta potential was measured using Zeta Probe, which adopts multiple frequency electroacoustic measurement technology from ColoidalDynamics, United States. To achieve accurate measurement, the standard solution was used to revise the conductivity, potential, pH value, and temperature before using the instrument. A total of 14 g grinded malachite samples was weighed at each experiment, quickly placed into the measuring cup, and combined with 275 mL deionized water. The stirring button was adjusted at a speed of 200 r/m, and 2 mol/L each of hydrochloric acid and sodium hydroxide solutions were used as titrants on the potentiometric titration.

### SEM–EDS analysis

After sulfidation and enhanced sulfidation, the surface morphology and composition of the malachite grains were studied through SEM and EDS using a Jeol JSM-6360 instrument at 20 kV.
